# Vascular Cambium: The Source of Wood Formation

**DOI:** 10.3389/fpls.2021.700928

**Published:** 2021-08-18

**Authors:** Dian Wang, Yan Chen, Wei Li, Quanzi Li, Mengzhu Lu, Gongke Zhou, Guohua Chai

**Affiliations:** ^1^College of Agronomy, Qingdao Agricultural University, Qingdao, China; ^2^College of Landscape Architecture and Forestry, Qingdao Agricultural University, Qingdao, China; ^3^State Key Laboratory of Tree Genetics and Breeding, Northeast Forestry University, Harbin, China; ^4^State Key Laboratory of Tree Genetics and Breeding, Chinese Academy of Forestry, Beijing, China; ^5^State Key Laboratory of Subtropical Silviculture, School of Forestry and Biotechnology, Zhejiang A&F University, Hangzhou, China; ^6^College of Resources and Environment, Qingdao Agricultural University, Qingdao, China

**Keywords:** wood, vascular cambium, hormones and peptides, cross talk regulation, *Arabidopsis* and *Populus*

## Abstract

Wood is the most abundant biomass produced by land plants and is mainly used for timber, pulping, and paper making. Wood (secondary xylem) is derived from vascular cambium, and its formation encompasses a series of developmental processes. Extensive studies in *Arabidopsis* and trees demonstrate that the initiation of vascular stem cells and the proliferation and differentiation of the cambial derivative cells require a coordination of multiple signals, including hormones and peptides. In this mini review, we described the recent discoveries on the regulation of the three developmental processes by several signals, such as auxin, cytokinins, brassinosteroids, gibberellins, ethylene, TDIF peptide, and their cross talk in *Arabidopsis* and *Populus*. There exists a similar but more complex regulatory network orchestrating vascular cambium development in *Populus* than that in *Arabidopsis*. We end up with a look at the future research prospects of vascular cambium in perennial woody plants, including interfascicular cambium development and vascular stem cell regulation.

## Introduction

**Figure 1:**
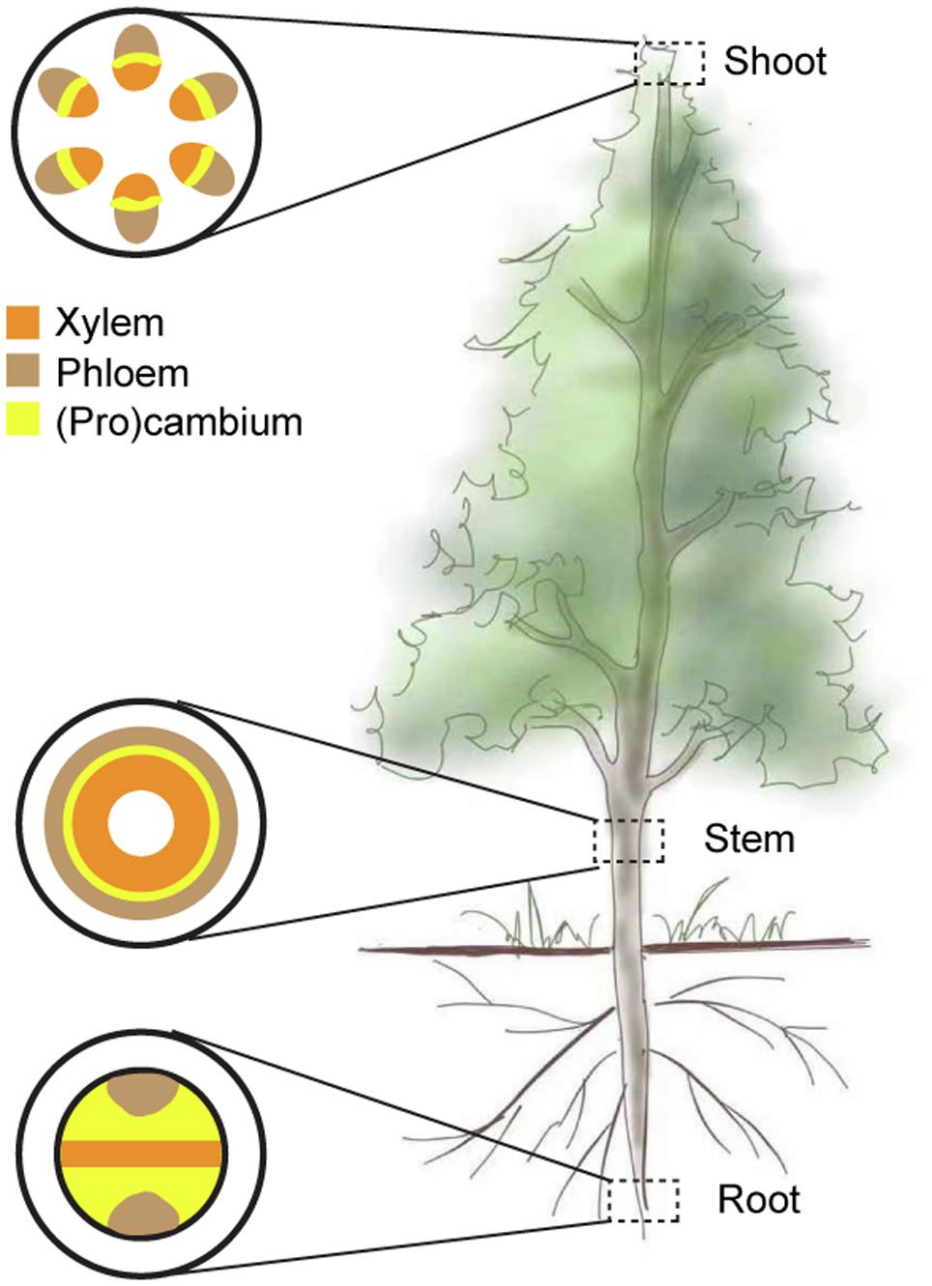
A schematic view of vascular tissue organization in shoot, stem, and root of vascular plants. Xylem and phloem tissues are initiated from procambium in vascular plants. Shoot apical meristems and root apical meristems are two primary meristems, and vascular cambium is a cylindrical secondary meristem in stems. The xylem is represented in orange, the phloem in brown, and the cambium in yellow.

Vascular plants, particularly tree species, undergo two distinct phases of growth and development. During primary growth, shoot apical meristems (SAMs) and root apical meristems (RAMs) are responsible for the aboveground and underground organ growth, respectively. At the peripheral region of SAM, procambium cells produce primary vascular bundles (Figure 1; also see Nieminen et al., 2015). After the primary vascular system is established, fascicular cambium located at the center of primary vascular bundles undergoes extension into the interfascicular region, forming a ring of vascular cambium (Figure 1; Nieminen et al., 2015). Vascular cambium is a cylindrical secondary meristem whose activity gives rise to the secondary growth. Like SAM and RAM, vascular cambium contains bifacial cambium stem cells in *Arabidopsis* (Shi et al., 2019; Smetana et al., 2019). However, stem cell activities of the three types of meristems are preferentially regulated by different members of the WUSCHEL-RELATED HOMEOBOX (WOX) and CLAVATA3/EMBRYO SURROUNDING REGION-RELATED (CLE) gene families: SAM is associated with WUSCHEL (WUS) and CLAVATA3 (CLV3; Mayer et al., 1998; Schoof et al., 2000), RAM with WOX5 and CLE40 (Sarkar et al., 2007; Berckmans et al., 2020), and vascular cambium with WOX4 and CLE41/44 (Hirakawa et al., 2010; Ji et al., 2010).

Secondary xylem (wood) and phloem are the inner and outer derivative products of the vascular cambium. Xylem is mainly comprised with dead cells with thickened cell walls rich in cellulose, hemicelluloses, and lignin and responsible for providing mechanical support and conducting water and minerals for the plant. Phloem transports photoassimilates and signaling molecules, including phytohormones and peptides, from the source organs to the sink organs. Fusiform initials and ray initials are morphologically distinct meristematic cells in vascular cambium of woody stems (Mauseth, 2016). The fusiform initials (>90% of the vascular cambium) are oriented longitudinally relative to the stem and undergo periclinal divisions that produce phloem and xylem mother cells (Mizrachi and Myburg, 2016; Fischer et al., 2019). The ray initials are isodiametric and produce the radially orientated ray cells that serve radial transport and storage.

**Figure 2:**
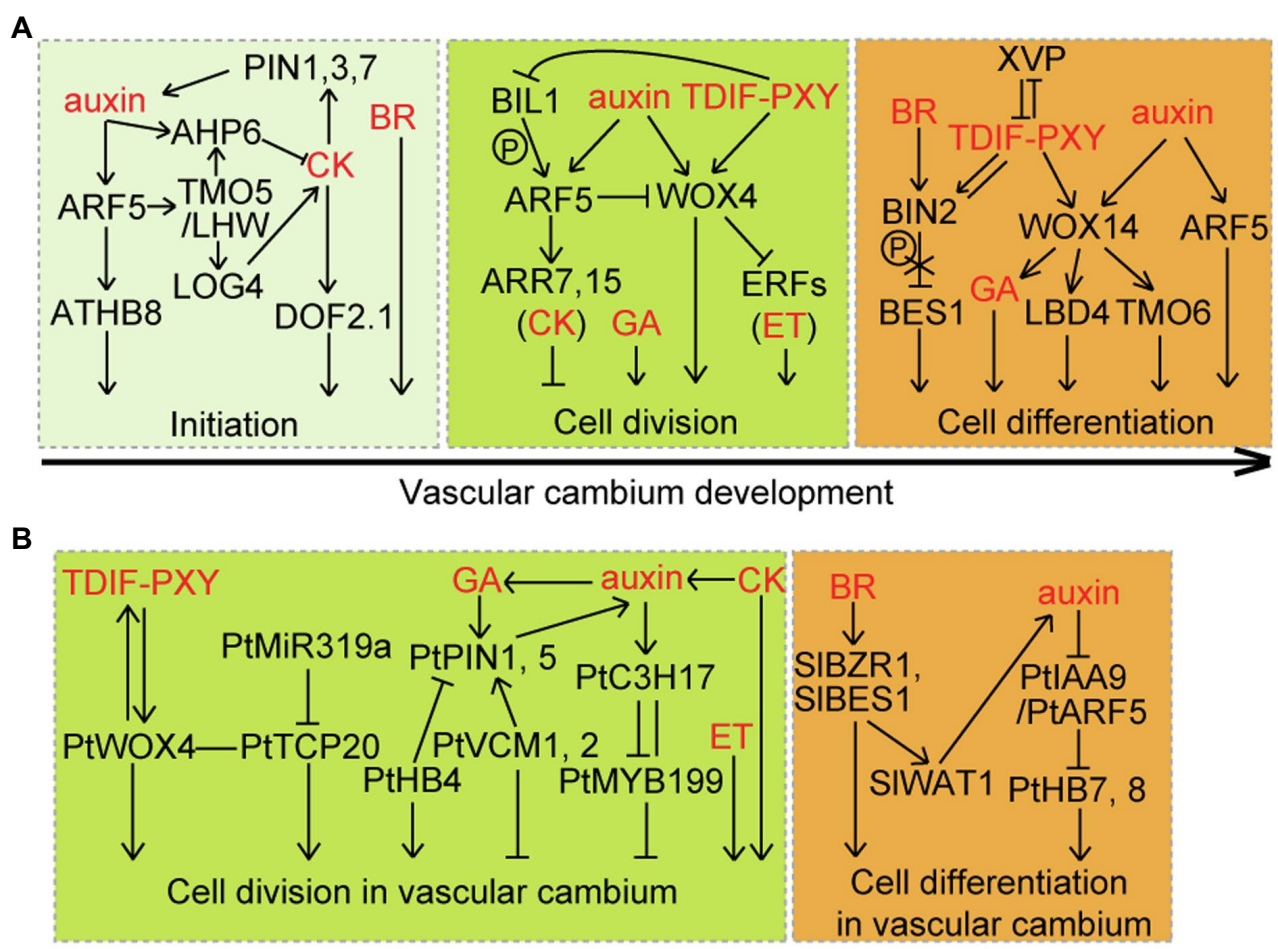
Coordination of multilayered signaling pathways on vascular cambium. **(A)** In *Arabidopsis*, vascular cambium initiation and the proliferation and differentiation of cambia derivative cells require a coordination of multiple signals, including auxin, cytokinin (CK), brassinosteroid (BR), gibberellin (GA), ethylene (ET), and TDIF-PXY. TDIF peptides are synthesized in the phloem and move to the cambium at which they bind to the PXY receptor. **(B)** Cross talk regulation of cell division and differentiation by multiple signals in the vascular cambium of *Populus* (Pt) and tomato (Sl) stems. There is a similar but more complex regulatory network orchestrating vascular cambium development in *Populus* than that in *Arabidopsis*.

The activity of the vascular cambium is regulated by endogenous developmental programs and environmental cues. In recent years, considerable progress in the molecular mechanism of the development of vascular cambium has been achieved in the model plants *Arabidopsis* and *Populus*. It has been shown that the establishment and maintenance of vascular cambium involve the coordination of multiple regulators, including hormones, peptides, and transcription factors (Figure 2; also see the reviews by Miyashima et al., 2013; Mizrachi and Myburg, 2016; Chiang and Greb, 2019). However, our knowledge about the development and regulation of vascular cambium, compared to SAM and RAM, is limited. This mini review focuses on recent progresses in the regulatory networks responsible for the vascular cambium identity and activity in poplar.

## Establishment of the Vascular Cambium

Because vascular procambial cells are imbedded under layers of other tissues in stems, our current understanding of procambium initiation and regulation is derived from studies in *Arabidopsis* embryos, RAMs, and leaf venation systems. Functional characterization of a serial of *Arabidopsis* mutants shows that vascular cambium initiation requires the cross talk regulation of multiple hormones (Figure 2A). Auxin plays a central role in regulating the initiation and maintenance of procambial stem cells (Ibañes et al., 2009; Weijers and Wagner, 2016). In pre-procambial strands, MONOPTEROS (MP)/AUXIN RESPONSE FACTOR 5 (ARF5) is activated in response to auxin and positively regulates the number of vascular initial cells through induction of the expression of the auxin efflux carrier gene *PIN-FORMED1* (*PIN1*; Wenzel et al., 2007). Periodic auxin maxima controlled by polar transport but not overall auxin levels is required to determine the radial pattern of vascular bundles in postembryonic growth (Ibañes et al., 2009). MP/ARF5 positively regulates TARGET OF MONOPTEROS 5 (TMO5), which forms a dimer complex with LONESOME HIGHWAY (LHW) to control the procambium cell divisions in roots (De Rybel et al., 2014; Ohashi-Ito et al., 2014). MP/ARF5 activates ATHB8-targeted PIN1 in response to auxin, forming a self-reinforcing mechanism of auxin flow during the formation of vein procambium (Donner et al., 2009). ATHB8, a HD-ZIP III transcription factor, is shown to restrict preprocambial cell specification to a narrow zone and stabilize preprocambial cell fate (Baima et al., 2001; Donner et al., 2009). *REVOLUTA* is another member of the *Arabidopsis* HD-ZIP III gene family, and its *Populus* ortholog, *PopREVOLUTA*, influences vascular cambium initiation in *Populus* stems (Robischon et al., 2011).

Cytokinin (CK) is another major hormone that regulates procambium identity and activity in *Arabidopsis* (Figure 2A). Mutation of three CK receptor genes *CYTOKININ RESPONSE 1* (*CRE1*), *ARABIDOPSIS HISTIDINE KINASE 2* (*AHK2*), and *AHK3* results in a severely reduced numbers of periclinal divisions in the procambium cells of the primary roots (Inoue et al., 2001). Accordingly, transgenic *Arabidopsis* plants overexpressing *CYTOKININ OXIDASES/DEHYDROGENASES 2* (*CKX2*), a CK degrading enzyme gene, under the control of *CRE1* promoter show the *cre1ahk2ahk3* phenotype (Mähönen et al., 2006). Moreover, the establishment of procambium cell identity requires a mutually inhibitory interaction between CK and auxin signaling (Figure 2A). Reduced CK signaling changes the subcellular polarity of PIN1, PIN3, and PIN7, while auxin is able to activate the expression of *ARABIDOPSIS HISTIDINE PHOSPHOTRANSFER 6* (*AHP6*), an inhibitor of CK signaling (Mähönen et al., 2006; Bishopp et al., 2011). Auxin-induced TMO5/LHW dimer directly activates *LONELY GUY 4* (*LOG4*) that encodes for a rate-limiting enzyme in CK biosynthesis (De Rybel et al., 2014). CK-dependent procambium cell divisions are controlled by the DOF transcription factor DOF2.1 downstream of TMO5/LHW (Smet et al., 2019).

Brassinosteroids (BRs) serve as a key promoting signal for procambial division during primary growth (Figure 2A). In the stem of *Arabidopsis*, the number of vascular bundles (VB) is obviously increased in gain-of-function BR-signaling mutants, such as *brassinosteroid insensitive 2* (*bin2*) and *brassinazole-resistant 1-1D* (*bzr1-1D*), while loss-of-function BR-signaling mutant *brassinosteroid insensitive 1-116* (*bri1-116*) and BR synthesis mutant *constitutive photomorphogenesis and dwarfism* (*cpd*) have fewer VBs than wild-type plants (Ibañes et al., 2009).

## Regulation of Vascular Cambium Activity

Trees display prominent secondary growth in the stem and root, with similar vascular cell types to *Arabidopsis* (Mizrachi and Myburg, 2016). Studies in *Arabidopsis* stems and roots indicate an important regulatory function for hormones (auxin, CK, and ethylene) and TRACHEARY ELEMENT DIFFERENTIATION INHIBITORY FACTOR (TDIF) peptide in the proliferation of vascular cambium (Ortega-Martinez et al., 2007; Matsumoto-Kitano et al., 2008; Suer et al., 2011; Fischer et al., 2019; Smetana et al., 2019). WOX4 is considered to be a central regulator of vascular cambium division (Figure 2A), because it activates a cambium-specific transcriptional network and integrates auxin, ethylene, and TDIF-PXY (PHLOEM INTERCALATED WITH XYLEM) signaling for cambium division (Hirakawa et al., 2010; Ji et al., 2010; Suer et al., 2011; Etchells et al., 2012; Brackmann et al., 2018; Zhang et al., 2019). WOX4 is required for auxin-dependent stimulation of cambium activity (Suer et al., 2011). Auxin-induced MP/ARF5 directly attenuates the activity of the stem cell-promoting *WOX4* gene, and cell-autonomously restricts the number of stem cells in stems (Brackmann et al., 2018). The TDIF peptides encoded by *CLE41* and *CLE44* are synthesized in the phloem and travel to the cambium where they bind and activate PXY, stimulating *WOX4* transcription and promoting cambium proliferation in stems (Hirakawa et al., 2010). Ethylene and TDIF signaling converge at WOX4 to regulate cambium activity (Etchells et al., 2012; Yang et al., 2020b). BIN2-LIKE 1 (BIL1), a glycogen synthase kinase 3, functions as a mediator that links auxin-CK signaling with TDIF-PXY signaling for the maintenance of cambial activity (Han et al., 2018). Phosphorylation of MP/ARF5 by BIL1 enhances its negative effect on the activity of vascular cambial, which upregulates ARABIDOPSIS RESPONSE REGULATOR 7 (ARR7) and ARR15, two negative regulators of CK signaling. BIL1 activity is inhibited by PXY, attenuating the effect of MP/ARF5 on *ARR7* and *ARR15* expressions and increasing vascular cambial activities.

Regulation of vascular cambium activity by auxin, CK, ethylene, and TDIF-PXY signaling is relatively conserved between trees and *Arabidopsis* (Figure 2). Auxin shows the highest level at the cambium zone, and its level declines near the mature xylem cells during wood formation in trees (Nilsson et al., 2008; Immanen et al., 2016). Overexpression of the stabilized form of INDOLE ACETIC ACID 3 (IAA3) that perturbs auxin signaling in hybrid aspen represses periclinal division of cambial cells but enlarges cell file harboring anticlinal cell division (Nilsson et al., 2008). Auxin-responsive *Pa*C3H17-*Pa*MYB199 module promotes cambium division by a dual regulatory mechanism in *Populus* stems (Tang et al., 2020). Auxin promotes direct repression of *PaMYB199* expression by *Pa*C3H17 and also enhances the *Pa*C3H17-*Pa*MYB199 interaction, attenuating *Pa*MYB199 inhibition of cambial cell division. Consistent with this, dominant repressors of *Pa*C3H17 or overexpression of *PaMYB199* result in a reduction in the number of cambial cell layers, while transgenic poplars overexpressing *PaC3H17* or repressing *PaMYB199* have the opposite phenotype. In addition, the regulation of vascular cambium activity is associated with feedback mediation of auxin homeostasis in trees. Downregulation of the *Populus* HD-ZIP III gene *PtrHB4* enhances *PtrPIN1* expression and causes drastic defects in interfascicular cambium, indicating that *Ptr*HB4 induces interfascicular cambium formation during the development of the secondary vascular system (Zhu et al., 2018). VASCULAR CAMBIUM-RELATED MADS 1 (VCM1) and VCM2 inhibit vascular cambium proliferation activity and secondary growth through direct upregulation of *PtrPIN5* expression in *Populus* stems (Zheng et al., 2021). These findings indicate more fine regulation of cambial activity by auxin signaling in trees than in *Arabidopsis*.

CK is another important regulator of cambial activity during wood formation (Figure 2B). Inhibition of cambial CK signaling by overexpression of *Arabidopsis AtCKX2* under the promoter of a birch *CRE1* gene leads to a reduced number of cambial cells in poplar stems, while increased vascular division is observed in transgenic poplars expressing the *Arabidopsis* CK biosynthetic gene *ISOPENTENYL TRANSFERASE 7* (*IPT7*) under the control of the cambium-specific *PttLMX5* promoter (Nieminen et al., 2008; Immanen et al., 2016). Elevated CK levels cause an increase of auxin level at the cambium zone, highlighting the interconnected nature of auxin and CK gradients (Immanen et al., 2016). A recent study uncovers the mechanism of CK signaling associated with its spatial enrichment to regulate vascular development in *Populus* (Fu et al., 2021). The local CK signaling in the developing secondary phloem regulates the activity of vascular cambium in a non-cell-autonomous manner.

In addition to auxin and CK, gibberellin (GA), ethylene, and TDIF-PXY signaling promote cambial cell division and radial growth in trees (Figure 2B). Transgenic poplar lines overexpressing *GA 20-OXIDASE*, encoding a GA biosynthesis enzyme, promote over-production of GA and cambium proliferation (Eriksson et al., 2000). Ethylene-overproducing and ethylene-insensitive poplars show increased and reduced cambium division, respectively (Love et al., 2009). Overexpression of *Ptt*CLE41, a TDIF-like peptide, together with its receptor *Ptt*PXYa affects the rate of cambial cell division and woody tissue organization in both hybrid aspen and poplar (Etchells et al., 2015; Kucukoglu et al., 2017). *Ptt*WOX4 stimulates the cambium proliferation downstream of TDIF-PXY signaling, as is similar to the manner of the *Arabidopsis* TDIF-PXY-WOX module. One difference is that in *Populus, PttWOX4a/b* expression is not responsive to auxin treatments, but upstream genes, such as *PttPXYa* and *PttCLE41a/d*, are responsive (Kucukoglu et al., 2017). The cross talk of hormones in regulation of cambium activity was also found in trees. For instance, GA coordinates with auxin for inducing cambium division through upregulating 83% of auxin-responsive genes, including *PttPIN1*, while auxin treatment upregulates GA biosynthesis genes and downregulates GA degradation genes in wood-forming tissues (Björklund et al., 2007).

## Regulation of Cambium Derivative Cells Differentiation

The regulatory roles of auxin, BR, and GA in cell differentiation in the vascular cambium are studied in *Arabidopsis* or/and trees (Figure 2). Since 20years ago, the IAA12/BODENLOS (BDL)-ARF5/MP module in auxin signaling has been identified to control provascular specification and patterning during embryo-genesis in *Arabidopsis* (Hardtke and Berleth, 1998). Recently, the *Pto*IAA9-*Pto*ARF5 module from *Populus* has been validated to mediate auxin-triggered cell differentiation of early developing xylem (Xu et al., 2019). With auxin treatment, *Pto*IAA9 protein is degraded, inducing *Pto*ARF5-activated gene expression, and the activated *PtoIAA9* switches-off auxin signaling in a self-controlled manner during wood formation. BRs play a regulatory role in differentiation of vascular tissues, in addition to inducing cambium initiation during primary growth. Mutation of both BRI-LIKE 1 (BRL1) and BRL3, two *Arabidopsis* vascular-specific BR receptors, causes expanded phloem development at the expense of xylem in stems (Cano-Delgado et al., 2004). *bri1-ethylmethylsulfone-suppressor 1-D* (*bes1-D*), a gain-of-function BR-signaling mutants, exhibits an increase of xylem differentiation (Kondo et al., 2014). Similarly, inhibition of BR synthesis results in decreased secondary vascular differentiation and cell wall biosynthesis, while elevated BR levels cause increases in secondary growth in *Populus* (Du et al., 2020). A recent study indicates that BR signaling is tightly connected with local intracellular auxin homeostasis during cell differentiation in the vascular cambium of tomato stems (Lee et al., 2021). BZR1/BES1-activated WALLS ARE THIN1 (WAT1), an auxin efflux carrier, facilitates cell differentiation in the vascular cambium by enhancing local auxin signaling. In addition, GA is shown to induce vascular cell differentiation and lignification downstream of *WOX14* gene in the stem of *Arabidopsis* (Mauriat and Moritz, 2009; Denis et al., 2017).

TDIF-PXY signaling is a mediator that induces cell differentiation in the vascular cambium in *Arabidopsis* (Figure 2A). Transgenic plants overexpressing *CLE41* or *CLE44* display abnormal vascular patterning with a xylem intermixed with phloem phenotype during both primary and secondary growths (Fisher and Turner, 2007; Etchells and Turner, 2010). TDIF signaling regulation of xylem differentiation is fine-tuned by the NAC transcription factor XYLEM DIFFERENTIATION AND ALTERED VASCULAR PATTERNING (XVP; Yang et al., 2020a). XVP negatively regulates the TDIF-PXY module, and it also forms a complex with TDIF co-receptors PXY-BAK1 (BRI1-associated receptor kinase 1). *XVP* expression is suppressed by TDIF by a feedback mechanism. Overexpression of *PttCLE41* or *PttPXY* (the orthologs to *Arabidopsis CLE41* and *PXY*, respectively) in hybrid aspen or poplar causes defects in the patterning of the vascular tissues and shows inhibited plant growth (Etchells et al., 2015; Kucukoglu et al., 2017), suggesting a similar regulation of xylem differentiation by the TDIF-PXY module in trees. The cross talk between TDIF-PXY signaling module and BR or auxin occurs in controlling vascular cell differentiation in *Arabidopsis* (Figure 2A). PXY physically interacts with BIN2 at the plasma membrane, and the treatments by TDIF peptide enhance the activity of BIN2 in a PXY-dependent manner (Kondo et al., 2014). Transcriptional regulatory network mediated by PXY comprises 690 transcription factor-promoter interactions, of which a feed-forward loop containing WOX14, TMO6 and their downstream gene LATERAL ORGAN BOUNDARIES DOMAIN4 (LBD4) determines the arrangement of vascular tissue (Smit et al., 2020).

The HD-ZIP III and NAC transcription factors are important regulators of vasculature organization. In *Arabidopsis* vascular tissues, mutation of one or several members of HD-ZIP III family results in an amphicribal vascular bundle pattern (phloem surrounding xylem), whereas gain-of-function mutants display amphivasal bundles (McConnell et al., 2001; Emery et al., 2003; Ramachandran et al., 2017). *PtrHB5* and *PtrHB7* are the orthologs of *Arabidopsis POPCORONA* and *AtHB8* in *Populus*, respectively. Both genes correspondingly induce cambium activity and xylem differentiation in stems during secondary growth (Du et al., 2011; Zhu et al., 2013). Interestingly, *PtrHB7* was identified as a direct target of the *Ptr*IAA9-*Ptr*ARF5 module during xylem cell differentiation (Xu et al., 2019). This places *Ptr*HB7 in the regulatory network of auxin-induced xylem differentiation in woody stems. The *Arabidopsis* NAC genes *VASCULAR-RELATED NAC DOMAINs* (*VNDs*) act as master regulators of xylem differentiation capable of switching on the developmental program (Kubo et al., 2005; Zhou et al., 2014), while other members of this family, *NAC SECONDARY WALL THICKENING PROMOTING FACTOR 1, 3* (*NST1, 3*), can promote fiber differentiation in stems (Zhong et al., 2006; Mitsuda et al., 2007). Four *Populus* orthologs of NST1/3 redundantly control SCW formation in xylem fibers, phloem fibers, and xylem ray parenchyma cells (Takata et al., 2019), indicating a conserved role of these NACs in wood formation. Some NAC genes impede xylem differentiation and secondary wall deposition involving *Pag*KNAT2/6b and *Pto*TCP20 in *Populus* (Hou et al., 2020; Zhao et al., 2020). *Pag*KNAT2/6b directly activates *PagXND1a* expression but represses *PagNST3s* and *PagVND6* expression in wood-forming tissues (Zhao et al., 2020). *Pto*TCP20 interacts with *Pto*WOX4a to control vascular cambium proliferation and also activates *PtoWND6* expression to promote secondary xylem differentiation (Hou et al., 2020).

## Future Outlook

Wood formation of tree species involves a complex regulatory network underlying cambial initiation, tissue patterning, and cell differentiation. Understanding the vascular cambium development is a basis for genetic modification of wood biomass and properties in trees. Extensive studies in the model tree *Populus* indicate the cross talk regulation of vascular cambium development by multiple signals, including auxin, CK, BR, and TDIF-PXY, similar to regulatory programs of *Arabidopsis* vascular development (Figure 2). However, based on genome sequences, it is predicted that 1.4~1.6 *Populus* homologs correspond to each *Arabidopsis* gene (Tuskan et al., 2006). These *Populus* duplicated genes may undergo divergent fates, such as nonfunctionalization (loss of original functions), neofunctionalization (acquisition of novel functions), or subfunctionalization (partition of original functions). This may explain the emerging more complex mechanisms underlying vascular cambium maintenance and differentiation in trees than in *Arabidopsis*.

In recent years, the studies on the vascular cambium formation and regulation in trees have been greatly facilitated by new technologies, such as the genome-editing, integrated-omics, and more advanced microscopy. Therefore, the following key questions are anticipated to be addressed in the near future.

1. How do the interfascicular cambial cells function in woody stems?

With the onset of the secondary growth, fascicular cambia are interconnected with interfascicular cambia located between the vascular bundles, forming a complete vascular cambium in woody stems (Figure 1). The interfascicular cambia are known to originate from the parenchymatic cells in the interfascicular region. Currently, our understanding regarding how the parenchymatic cells differentiate and develop into new procambium strands in the interfascicular region is limited, compared with extensive studies on fascicular cambia. To our knowledge, the HD-ZIP III gene *PtrHB4* is the only gene that is shown to induce interfascicular cambium division in *Populus* stems (Zhu et al., 2018). Analysis of time-spatial features of parenchymatic cells action and mining the related genes in trees are essential in the future. The application of single-cell RNA sequencing, computational modeling, or biosensor may be helpful for addressing this question.

2. How is the vascular cambium activity maintained in trees?

Vascular cambium of trees is able to ensure both increased stem girth and annual renewal of vascular tissues over its lifespan. Even in 667-year-old *Ginkgo biloba* trees, the vascular cambium still maintains activity (Wang et al., 2020). A key question for wood biology is how vascular cambium activity maintained? In *Populus*, multiple signals mediate the coordinated regulation of vascular cambium activity, as is more complex than that in *Arabidopsis* (Figure 2). It is therefore critical to investigate what signals and how these signals drive the activity of cambial stem cells under certain circumstances? Identification of reliable cell-specific makers thus to analyze gene expression in each layer of cambial cells is essential for understanding the gene regulation of vascular stem cells in trees.

## Author Contributions

DW and GC drafted the manuscript. GZ, YC, WL, QL, and ML edited the manuscript. All authors approved the final version.

## Conflict of Interest

The authors declare that the research was conducted in the absence of any commercial or financial relationships that could be construed as a potential conflict of interest.

## Publisher’s Note

All claims expressed in this article are solely those of the authors and do not necessarily represent those of their affiliated organizations, or those of the publisher, the editors and the reviewers. Any product that may be evaluated in this article, or claim that may be made by its manufacturer, is not guaranteed or endorsed by the publisher.
